# Supplementary material for: Seed-borne bacteria drive wheat rhizosphere microbiome assembly via niche partitioning and facilitation
Source: Nat Microbiol. 2025 Mar 26;10(5):1130–44. doi: 10.1038/s41564-025-01973-1 (PMC12055584; doi:10.1038/s41564-025-01973-1)
Supplement: Supplementary file 1 — Supplementary Figs. 1–8. [file 41564_2025_1973_MOESM1_ESM.pdf]

nature microbiology

Supplementary information https://doi.org/10.1038/s41564-025-01973-1

Seed-borne bacteria drive wheat rhizosphere microbiome assembly via niche partitioning and facilitation

In the format provided by the authors and unedited

Supplementary figures

Supplementary Fig. 1

Supplementary Fig. 2

Supplementary Fig. 3

Supplementary Fig. 4

Supplementary Fig. 5

Supplementary Fig. 6

Supplementary Fig. 7

Supplementary Fig. 8

Supplementary figures

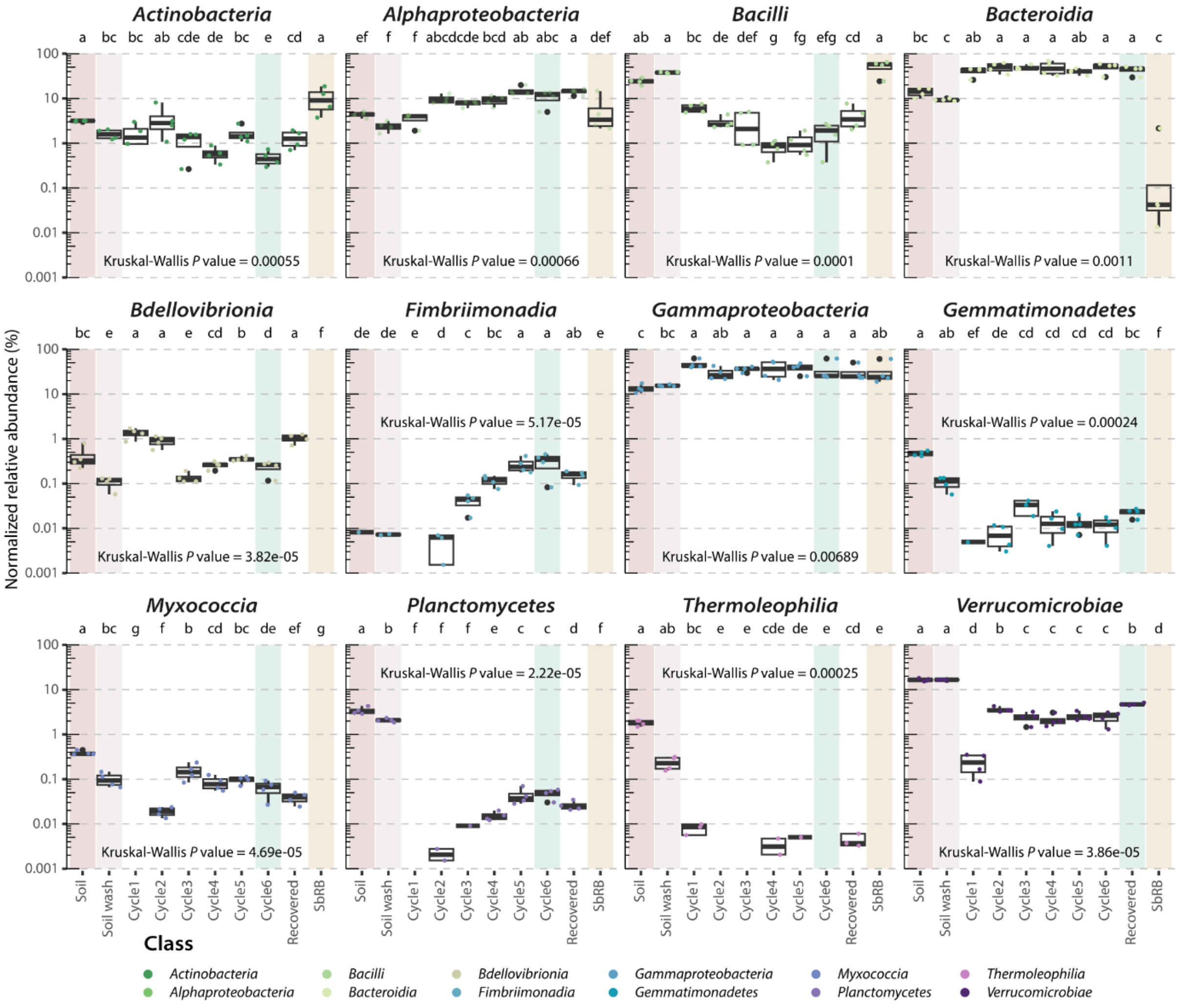

Supplementary Fig. 1 | Differences in relative abundance of bacterial classes across samples. CSS-normalized relative abundance of the top bacterial classes across samples. Letters denote different statistically significant groups (P value < 0.05) using Kruskal-Wallis rank sum test with LSD post hoc analysis and P value corrected by fdr. Dots represent individual replicates (n = 4) per sample. The center line shows the median, the box spans the first to third quartiles, and whiskers extend to 1.5× the interquartile range. Outliers beyond this are shown as individual dots.

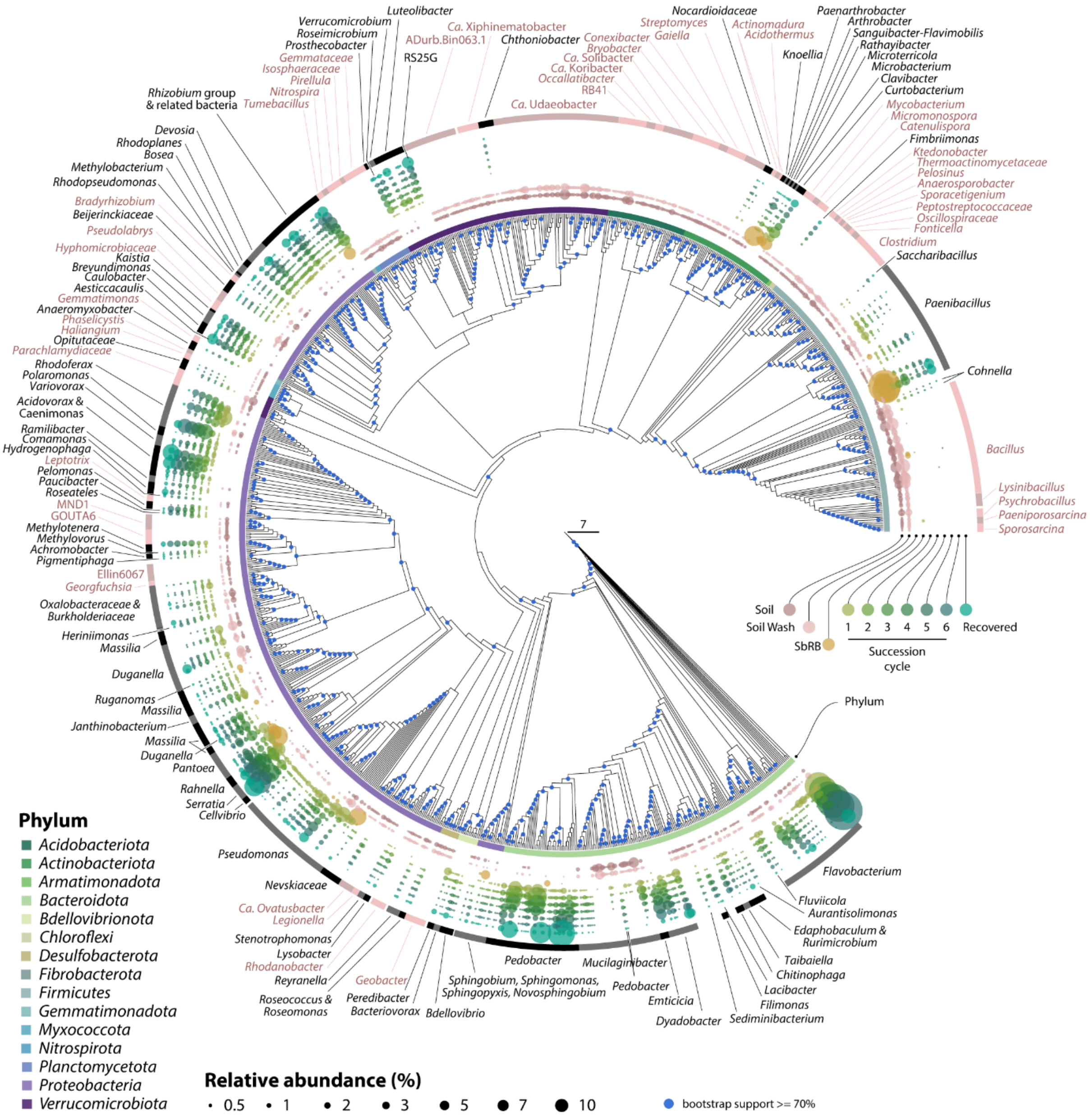

Supplementary Fig. 2 | Selection of ASVs during the succession of the wheat rhizobiome. The phylogenetic tree was built using ASVs with a total mean (*n* = 4) relative abundance ≥ 0.005%. Names of relevant genera selected (black/grey) or not (pink/dark pink) after the succession are indicated. From inwards to outwards, colored dots represent ASVs, with sizes corresponding to their relative abundance in soil, soil wash, SbRB, succession cycles and the recovered rhizosphere communities.

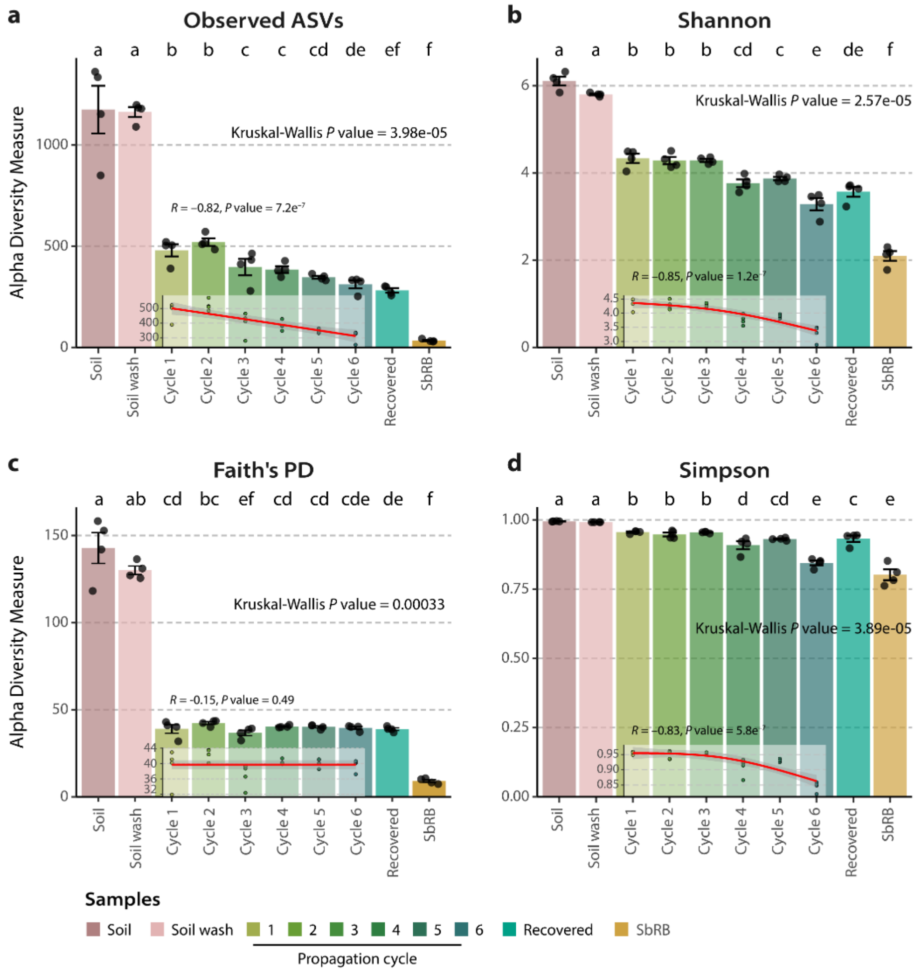

Supplementary Fig. 3 | Alpha diversity of samples across succession cycles. Number of observed ASVs (**a**), Shannon diversity (**b**), Faith's phylogenetic diversity (PD, **c**), and Simpson diversity (**d**) across samples based on 16S rRNA amplicon sequence variants. Alpha diversity measures are indicated as bars (mean values) with the standard error. Black dots indicate values of individual replicates (n = 4). Letters denote different statistically significant groups (P value < 0.05) using Kruskal-Wallis rank sum test with LSD post hoc analysis and P value corrected by fdr. Spearman correlation of each alpha diversity measure and succession cycle is shown within the plots. Correlation coefficient (R) and P value are indicated. Curves represent the general additive model fit (mean, red line) and the 95% confidence interval (shaded).

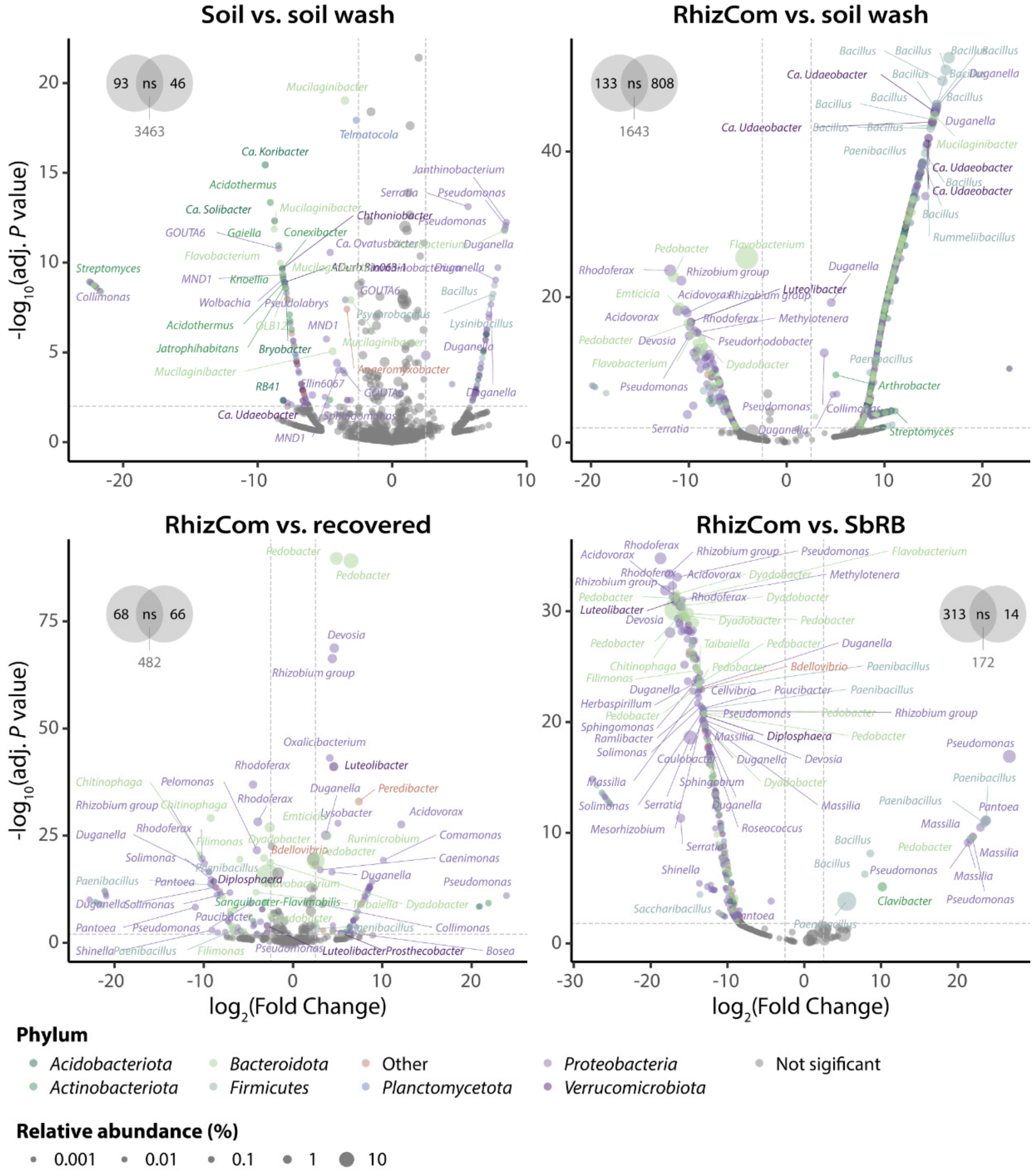

Supplementary Fig. 4 | Differential abundance of 16S RNA amplicon sequence variants. Differential abundance of ASVs calculated with DESeq2 and using two-sided Wald test with a local estimate of dispersion. ASVs with a |log$_2$(fold change) ≥ 2.5 and an adjusted (adj.) *P* value < 0.01 were considered as significant. Dots represent ASVs, colored according to their phylum and sized according to their relative abundance. ASVs with a relative abundance ≥ 0.05 were named at the genus level, up to a maximum of 45 label overlaps.

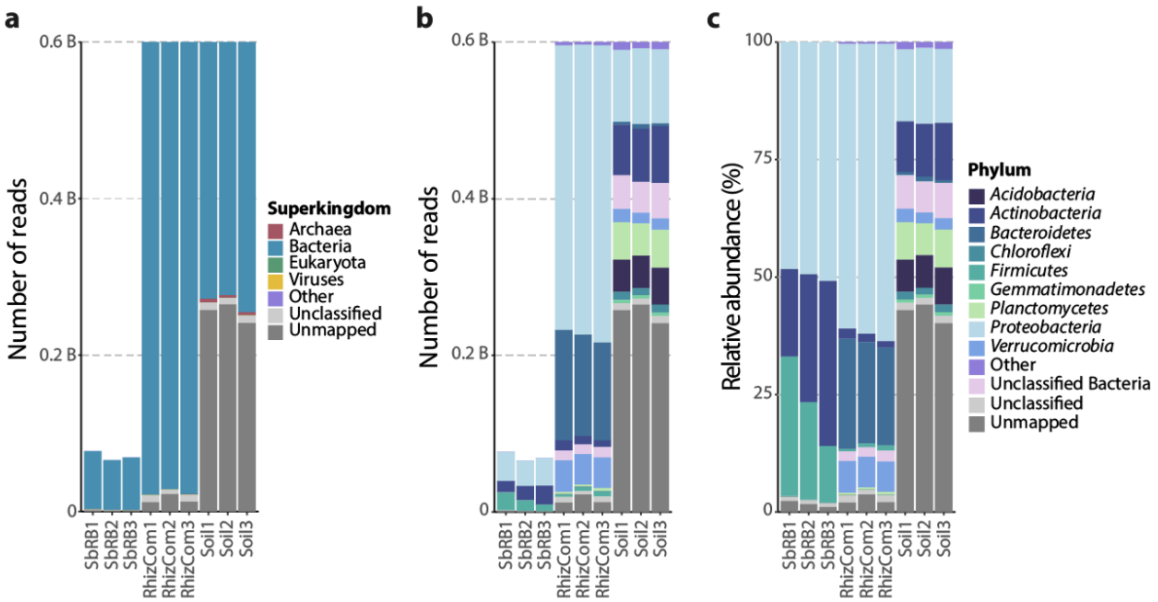

Supplementary Fig. 5 | Taxonomic classification of reads in the metagenome samples. a, Total number of reads mapping to coding DNA sequences and their taxonomic assignment at the Superkingdom level. **b**, Total number or **c**, relative abundance of reads assigned to Bacteria colored according to their phylum. Bars represent mean values (*n* = 3).

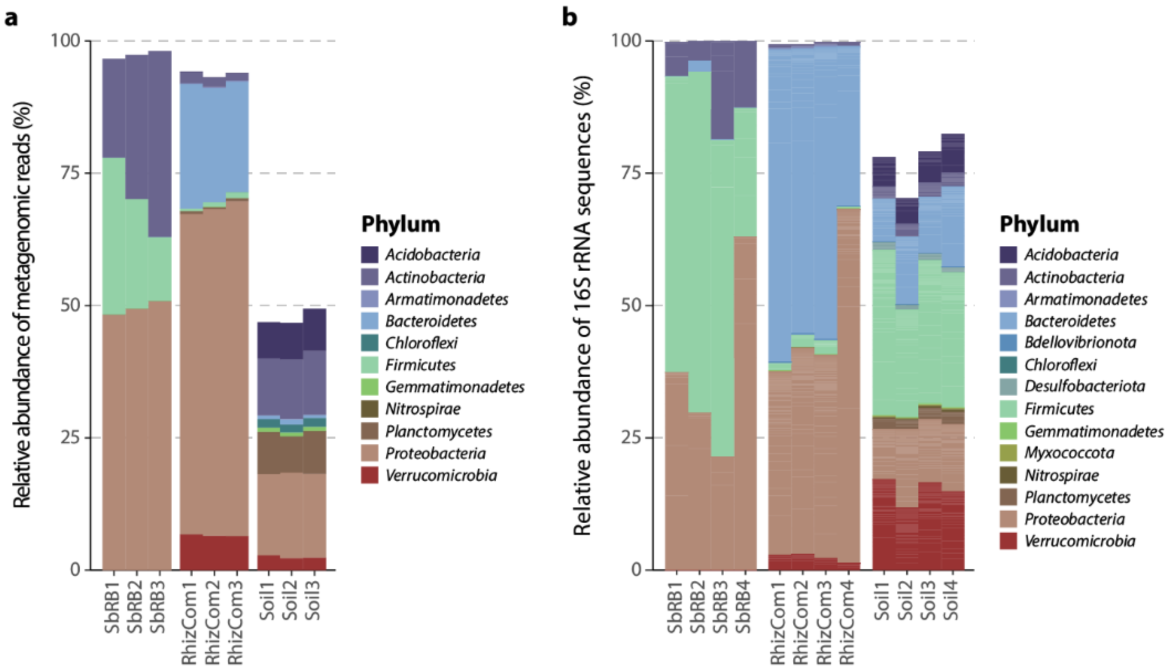

Supplementary Fig. 6 | Concordance between taxonomic classification of metagenomic samples vs. samples characterized by 16S rRNA amplicon sequencing. a, Relative abundance of metagenomic reads at the phylum level. Bars represent mean values (*n* = 3) **b**, Relative abundance of 16S rRNA sequences at the phylum level. Only the top 1,000 ASVs are shown. Bars represent mean values (*n* = 4).

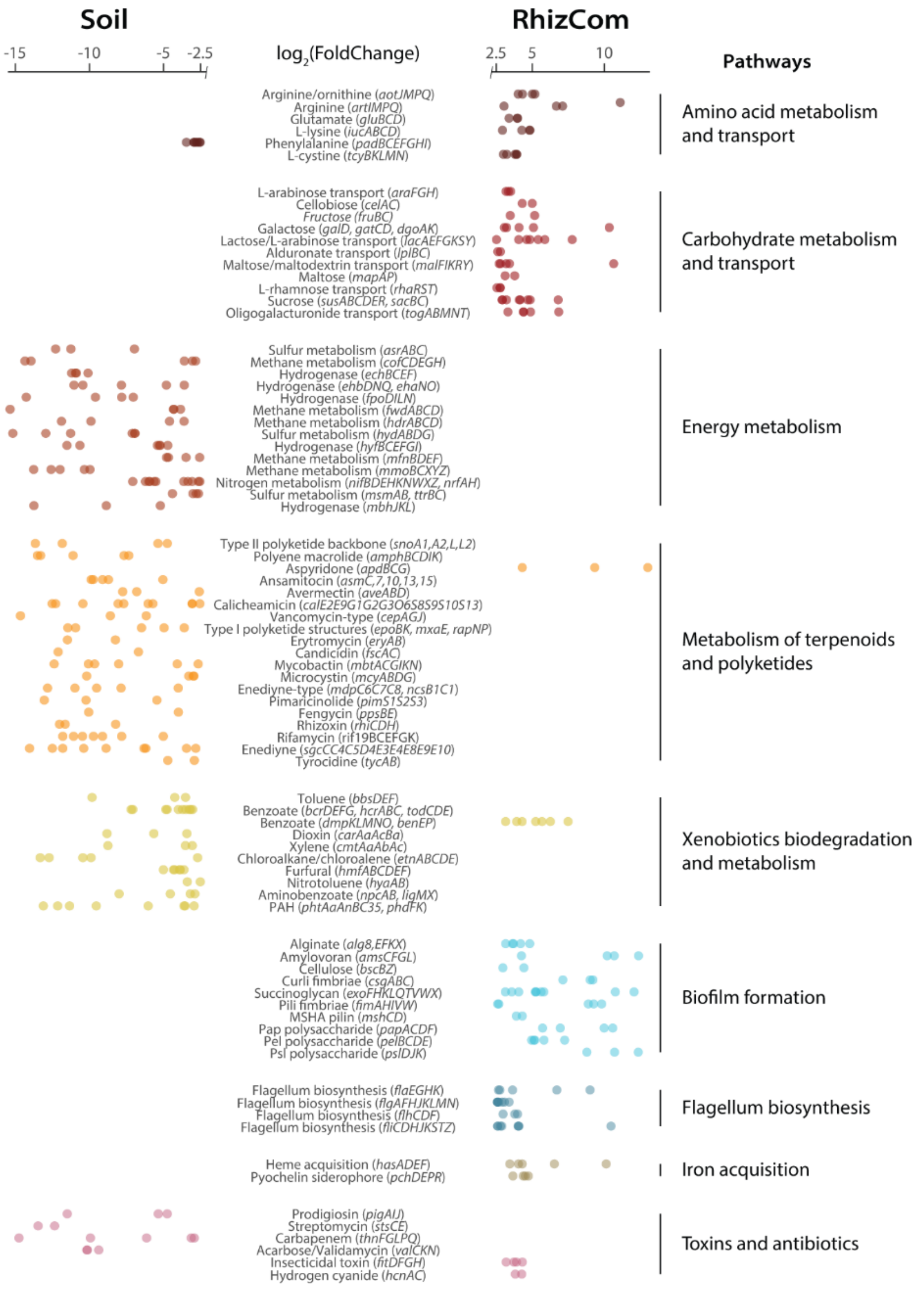

Supplementary Fig. 7 | Differential abundance of gene clusters between the RhizCom and soil. Differential abundance of KEGGs annotations between the soil and the RhizCom. Results were filtered to show only significantly enriched annotations (adjusted P value ≤ 0.001 and $|\log2(\text{fold change})| \geq 2.5$) and those annotations that were part of a gene cluster in which multiple genes were differentially abundant. Putative function and gene names are indicated.

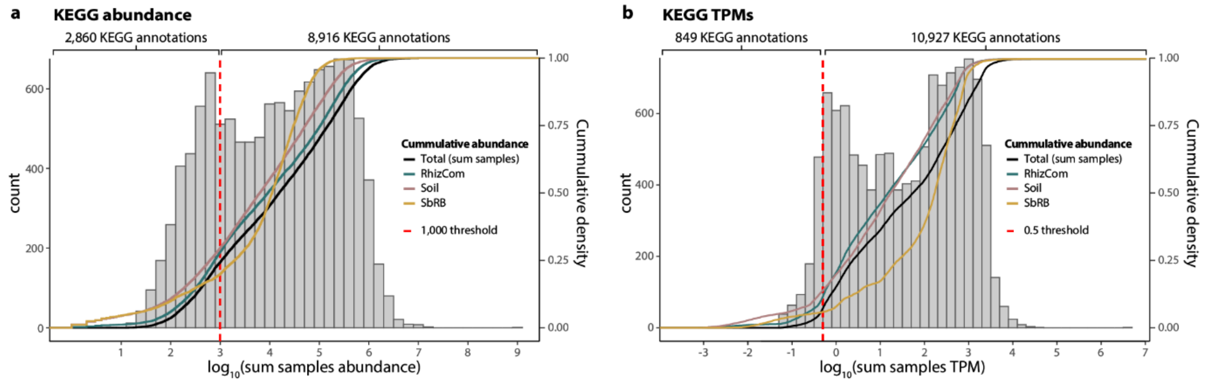

Supplementary Fig. 8 | Thresholds of abundance and TPMs for KEGG annotations. ab, Histograms (bars, left axis) and empirical cumulative distributions (lines, right axis) of KEGG annotations based on abundance (**a**) and TPMs (**b**). The black line represents the sum of abundances for all samples, while colored lines represent the sum of abundances for the replicates of a sample. The dashed red line indicates an abundance threshold value ≥ 1,000 for KEGG abundances (**a**) or ≥ 0.5 for KEGG TPMs (**b**) used in this study. Numbers above the plot indicate the KEGG annotations above or below the chosen threshold.
