# Supplementary material for: Seed-borne bacteria drive wheat rhizosphere microbiome assembly via niche partitioning and facilitation
Source: Nat Microbiol. 2025 Mar 26;10(5):1130–44. doi: 10.1038/s41564-025-01973-1 (PMC12055584; doi:10.1038/s41564-025-01973-1)
Supplement: Supplementary file 2 — Reporting Summary [file 41564_2025_1973_MOESM2_ESM.pdf]

nature portfolio

Corresponding author(s): Daniel Garrido-Sanz, Christoph Keel

Last updated by author(s): Feb 18, 2025

Reporting Summary

Nature Portfolio wishes to improve the reproducibility of the work that we publish. This form provides structure for consistency and transparency in reporting. For further information on Nature Portfolio policies, see our Editorial Policies and the Editorial Policy Checklist.

Statistics

For all statistical analyses, confirm that the following items are present in the figure legend, table legend, main text, or Methods section.

n/a	Confirmed	
☐	☒	The exact sample size (*n*) for each experimental group/condition, given as a discrete number and unit of measurement
☐	☒	A statement on whether measurements were taken from distinct samples or whether the same sample was measured repeatedly
☐	☒	The statistical test(s) used AND whether they are one- or two-sided<br>*Only common tests should be described solely by name; describe more complex techniques in the Methods section.*
☒	☐	A description of all covariates tested
☐	☒	A description of any assumptions or corrections, such as tests of normality and adjustment for multiple comparisons
☐	☒	A full description of the statistical parameters including central tendency (e.g. means) or other basic estimates (e.g. regression coefficient) AND variation (e.g. standard deviation) or associated estimates of uncertainty (e.g. confidence intervals)
☐	☒	For null hypothesis testing, the test statistic (e.g. *F*, *t*, *r*) with confidence intervals, effect sizes, degrees of freedom and *P* value noted<br>*Give P values as exact values whenever suitable.*
☒	☐	For Bayesian analysis, information on the choice of priors and Markov chain Monte Carlo settings
☒	☐	For hierarchical and complex designs, identification of the appropriate level for tests and full reporting of outcomes
☒	☐	Estimates of effect sizes (e.g. Cohen's *d*, Pearson's *r*), indicating how they were calculated

Our web collection on statistics for biologists contains articles on many of the points above.

Software and code

Policy information about availability of computer code

Data collection

Software used: Microsoft Excel (version 2409 Build 16.0.18025.20160).

Data analysis

All the analyses were performed on a Dell workstation with an Intel(R) Xeon(R) Gold 6248R CPU processor, 384 GB of RAM, and Windows 10 Pro operating system.

Metagenome analyses were performed in a Linux environment, using Ubuntu 18.04 distribution, operating on the Windows Subsystem for Linux (version 2.3.26.0). The software used were fastp (version 0.23.2), bowtie2 (version 2.5.1), SAMtools (version 1.7), Nonpareil3 (version 3.401), BBnorm (version 37.62), Seqkit (version 2.2.0), MEGAHIT (version 1.2.9), QUAST (version 5.2.0), SqueezeMeta (version 1.6.2), diamond (version 2.0.15.153), Prodigal (v2.6.3), HMMER (v3.4), CONCOCT (version 1.1.0), MetaBAT 2 (version 1:2.15), MaxBin (version 2.2.6), DASTool (version 1.1.1), CheckM (version1.1.6), PhyloPhlAn (version 3.0.67) and RAxML (version 8.2.12)

Data processing, plotting and statistical analyses were performed using R (version 4.1.1 or 4.4.1) on RStudio (version 2024.04.2 Build 764). The packages used were agricolae (version 1.3-7), ape (version 5.8), Biostrings (version 2.70.3), car (version 3.1-3), circlize (version 0.4.16), ComplexHeatmap (version 2.20.0), dada2 (version 1.30.0), data.table (version 1.15.4), DECIPHER (version 2.30.0), DESeq2 (version 1.44.0), dplyr (version 1.1.4), ggalluvial (version 0.12.5), ggforce (0.4.2), ggh4x (version 0.2.8), ggnewscale (version 0.5.0), ggplot2 (version 3.5.1), ggplotify (version 0.1.2), ggpmisc (version 0.6.0), ggpubr (version 0.6.0), ggrepel (version 0.9.5), ggsci (version 3.2.0), ggtern (version 3.5.0), ggtree (version 3.12.0), ggtreeExtra (version 1.14.0), gridExtra (version 2.3), KEGGREST (version 1.44.1), metagenomeSeq (version 1.43.0), metagMisc (version 0.5.0), Nonpareil (version 3.5.3), pairwiseAdonis (version 0.4.1), pals (version 1.9), pathview (version 1.44.0), phangorn (version 2.11.1), phyloseq (version 1.46.0), phyloseq.extended (version 0.1.0.9000), picante (version 1.8.2), plotly (version 4.10.4), qiime2R (version 0.99.6), ranacapa (version0.1.0), RColorBrewer (version 1.1-3), scales (version 1.3.0), scico (version 1.5.0), seqinr (version 4.2-36), speedyseq (version 0.5.3.9021), SQMtools (version 1.6.3), stats (version 3.1-3) tidyverse (version 2.0.0) and vegan (version 2.6-6.1).

Data used for statistical analyses were tested using the Saphiro-Wilk normality test, and the Levene's test for homogeneity of variances. Data that did not have a normal distribution or homogeneous variances were analyzed with the agricolae v1.4-5 R package, using the non-parametric Kruskal-Wallis rank sum test, with Fisher's least significant difference (LSD) post hoc criterium, and correction of P values using the false discovery rate (fdr). Pairwise comparisons were performed using two-sided Wilcoxon rank-sum tests within the stats v4.4.1 R package. For testing increases in bacterial growth when co-cultured with helper strains, one-sided Wilcoxon rank-sum tests were used, with the "greater" alternative hypothesis. Data that followed a normal distribution and with homogeneous variances were analyzed using PERMANOVA (permutational multivariate analyses of variance) or ANOSIM (analysis of similarity), using with the adonis2 or anosim functions, respectively, within the vegan v1.5-4 R package, and using 9999 permutations. Spearman correlations were calculated using the stat_cor function within the ggpubr R package, and data was fitted to a general additive model (GAM), using a k = 3 with the geom_smooth ggplot2 v3.5.1 R function, and a confidence interval of 0.95. No statistical method was used to predetermine sample size.

The code used for the analysis of the 16S rRNA gene amplicon data, metagenomic shotgun data, and other analyses reported in this study is available on GitHub (https://github.com/dgarrs/RhizCom) and Zenodo (https://doi.org/10.5281/zenodo.13969370).

For manuscripts utilizing custom algorithms or software that are central to the research but not yet described in published literature, software must be made available to editors and reviewers. We strongly encourage code deposition in a community repository (e.g. GitHub). See the Nature Portfolio guidelines for submitting code & software for further information.

Data

Policy information about availability of data

All manuscripts must include a data availability statement. This statement should provide the following information, where applicable:

- Accession codes, unique identifiers, or web links for publicly available datasets
- A description of any restrictions on data availability
- For clinical datasets or third party data, please ensure that the statement adheres to our policy

Raw reads from both 16S rRNA gene amplicons and metagenomic shotgun have been deposited in the NCBI Sequence Read Archive (RSA) database and are publicly available under the BioProject accession number PRJNA1169405. The 16S rRNA gene sequences of the five isolated bacterial strains reported in this work have been submitted to the NCBI GenBank database under the following accession numbers: Variovorax sp. DGS2, PQ776219; Acidovorax sp. DGS4, PQ776220; Pseudomonas sp. DGS16, PQ776221; Paenibacillus sp. DGS31, PQ776222; and Pantoea sp. SbRB3, PQ776223. Other raw data generated in this study are provided in the Supplementary information or in the GitHub repository https://github.com/dgarrs/RhizCom, and available on Zenodo under https://doi.org/10.5281/zenodo.13969370.

Databases and datasets used in this study are available as follows. Wheat reference genome (Triticum aestivum, NCBI GenBank acc. no. GCA_903993985.1). SILVA database v138.1 (https://www.arb-silva.de/documentation/release-138.1/). Databases GeneBank nr, eggNOG, KEGG and Pfam were downloaded using the script make_databases.pl from SqueezeMeta. PhyloPhlAn database of 400 universal amino acid marker sequences was downloaded using the phylophlan_setup_database script of PhyloPhlAn. All databases were downloaded in May 2023.

Research involving human participants, their data, or biological material

Policy information about studies with human participants or human data. See also policy information about sex, gender (identity/presentation), and sexual orientation and race, ethnicity and racism.

Reporting on sex and gender	N/A.
Reporting on race, ethnicity, or other socially relevant groupings	N/A.
Population characteristics	N/A.
Recruitment	N/A.
Ethics oversight	N/A.

Note that full information on the approval of the study protocol must also be provided in the manuscript.

Field-specific reporting

Please select the one below that is the best fit for your research. If you are not sure, read the appropriate sections before making your selection.

☒ Life sciences ☐ Behavioural & social sciences ☐ Ecological, evolutionary & environmental sciences

For a reference copy of the document with all sections, see nature.com/documents/nr-reporting-summary-flat.pdf

Life sciences study design

All studies must disclose on these points even when the disclosure is negative.

Sample size	All sample sizes are described in the Method section of the paper. Microbiome (16S rRNA amplicon sequencing) samples were obtained as a pool of 4 individual plant rhizospheres. Per sample, 4 pools were obtained and sequenced. For metagenomes (shotgun illumina sequencing), samples were as above, but only 3 out of the 4 pools were sequenced. The choice of samples to be sequenced by shotgun was based on the most apparent similar ones in terms of amplicon profiling. The selected sample sizes provide sufficient number of replicates for meaningful ecological comparisons while balancing sequencing costs and data quality.
Data exclusions	No data were excluded for microbiome analysis (16S rRNA amplicon sequencing). For metagenomes (shotgun illumina sequencing), one out of the four replicate pools per sample were excluded based on the most apparent dissimilar sample in terms of amplicon profiling.
Replication	All samples contained at least 3 replicates, product of the pooling of four plant rhizospheres. All replication attempts were successful.
Randomization	N/A for this study. Sample collection and processing was driven by experimental design rather than random assignment. Microbiome and metagenome samples were obtained from plant rhizospheres, where pooling was necessary to capture biological variability. In addition, metagenome sequencing was performed on samples selected based on similarity of amplicon profiles to ensure representative shotgun sequencing. Given these constraints, randomization was neither practical nor relevant to the study objectives.
Blinding	Blinding was not possible for this study. The investigator that collected the data also performed the analyses.

Reporting for specific materials, systems and methods

We require information from authors about some types of materials, experimental systems and methods used in many studies. Here, indicate whether each material, system or method listed is relevant to your study. If you are not sure if a list item applies to your research, read the appropriate section before selecting a response.

Materials & experimental systems

n/a	Involved in the study	
☒	☐	Antibodies
☒	☐	Eukaryotic cell lines
☒	☐	Palaeontology and archaeology
☒	☐	Animals and other organisms
☒	☐	Clinical data
☒	☐	Dual use research of concern
☐	☒	Plants

Methods

n/a	Involved in the study	
☒	☐	ChIP-seq
☒	☐	Flow cytometry
☒	☐	MRI-based neuroimaging

Plants

Seed stocks	Non-treated Triticum aestivum var. Arina seeds (Lot number 111.1001) were obtained from Delley seeds and plants Ltd, Switzerland, on March 2021.
Novel plant genotypes	N/A.
Authentication	Autentification is carried out by seed bank supplier. The variety is registered in the National Swiss Catalog (CN 1981), and the List of Recommended Varieties (LR 1981)
